# Dynamic maximum entropy provides accurate approximation of structured population dynamics

**DOI:** 10.1371/journal.pcbi.1009661

**Published:** 2021-12-01

**Authors:** Katarína Bod’ová, Enikő Szép, Nicholas H. Barton

**Affiliations:** 1 Faculty of Mathematics, Physics and Informatics, Comenius University, Bratislava, Slovakia; 2 Institute of Science and Technology Austria (IST Austria), Klosterneuburg, Austria; University of California Riverside, UNITED STATES

## Abstract

Realistic models of biological processes typically involve interacting components on multiple scales, driven by changing environment and inherent stochasticity. Such models are often analytically and numerically intractable. We revisit a dynamic maximum entropy method that combines a static maximum entropy with a quasi-stationary approximation. This allows us to reduce stochastic non-equilibrium dynamics expressed by the Fokker-Planck equation to a simpler low-dimensional deterministic dynamics, without the need to track microscopic details. Although the method has been previously applied to a few (rather complicated) applications in population genetics, our main goal here is to explain and to better understand how the method works. We demonstrate the usefulness of the method for two widely studied stochastic problems, highlighting its accuracy in capturing important macroscopic quantities even in rapidly changing non-stationary conditions. For the Ornstein-Uhlenbeck process, the method recovers the exact dynamics whilst for a stochastic island model with migration from other habitats, the approximation retains high macroscopic accuracy under a wide range of scenarios in a dynamic environment.

## Author summary

Complex processes in biology and physics have much in common. Collective motion of animals can be well described by models of interacting particles and emergent collective behavior can often be characterized as phase transitions of such a model. When the system is settled to a steady state, statistical physics connects random fluctuations of the process with key macroscopic quantities, using the maximum entropy method. It is thus not surprising that this method is in turn useful in understanding biological systems. However, realistic problems such as structured population dynamics, studied in our work, are set in dynamic environments, caused for instance by fluctuations in food supply. Therefore, we use the dynamical maximum entropy approximation, which allows us to reduce the full problem to simpler dynamics, without the need to track microscopic details. We focus on two processes (one in physics, one in biology) in our study: the motion of a charged particle and the dynamics of structured populations, both in a changing environment. We show that the method is extremely accurate even when the environmental changes are very fast, thus providing a powerful tool to study both biological and physical processes in changing environments.

## Introduction

Conceptual understanding of realistic problems in applied sciences is often hindered by the curse of complexity, with quantities of interest coupling to finer features. Due to their multiscale character even simple questions lead to exploration of the full complexity of the system. But how can we ever understand the processes around us if incremental learning is impossible?

**Fig 1:**
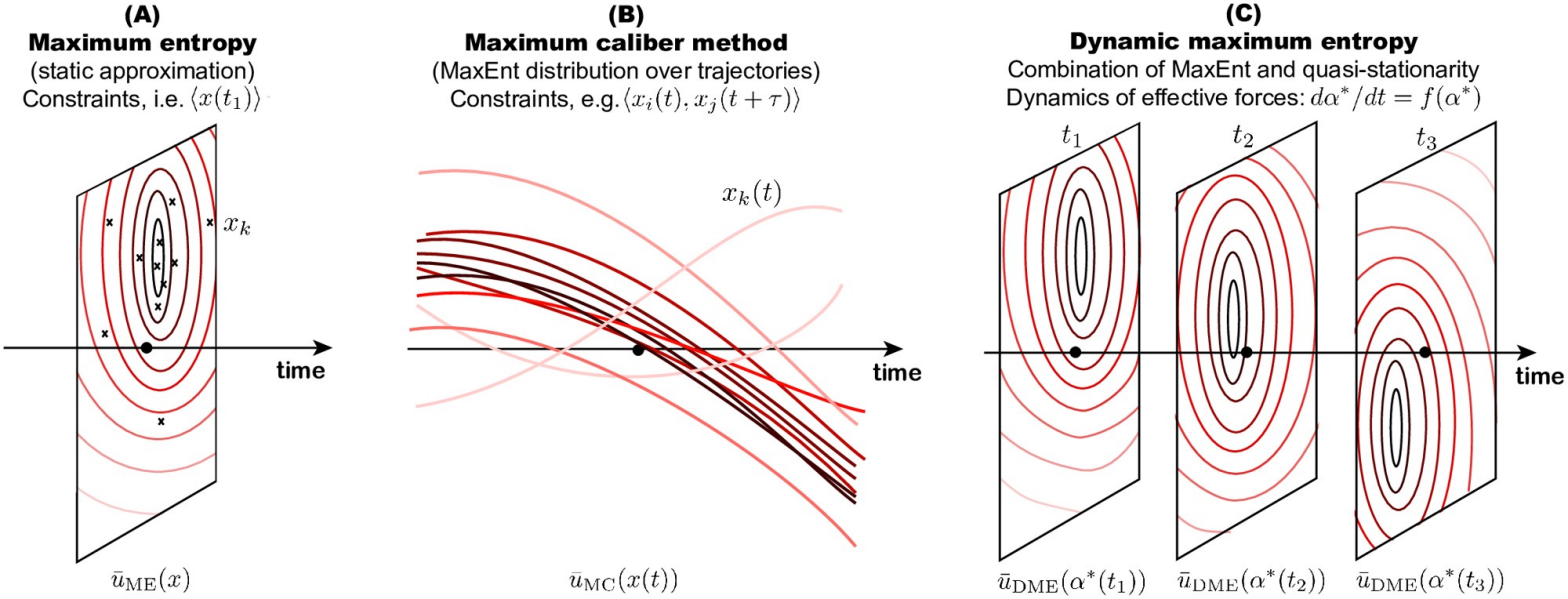
Variational methods ME and MC compared to DME. (A) ME looks at a snapshot *x* of a process at a particular time and provides an approximation ${\bar{u}}_{\text{ME}}\left(x\right)$ of the microscopic distribution, given knowledge of a few key macroscopic observables. (B) MC is analogous to ME, however, each data point represents a trajectory *x*(*t*). MC connects the microscopic distribution over possible trajectories with macroscopic constraints and approximates it by ${\bar{u}}_{\text{MC}}\left(x\left(t\right)\right)$. (C) DME is a quasi-stationary approximation of the stochastic dynamics, given by the FPE, which reduces the full problem to a low-dimensional dynamics. This reduction is a consequence of a ME ansatz; the approximation at each time ${\bar{u}}_{\text{DME}}\left(\alpha \left(t\right)\right)$ solves the ME problem (stationary form in the FPE), where the dynamics of the effective forces ***α*** are systematically derived from the FPE.

Statistical mechanics provides a clever way to understand complex multiscale problems by linking processes on different scales through the parsimony principle—the method of maximum entropy (ME), introduced by [1]. ME has the form of a variational problem where an entropy of the microscopic distribution is maximized, while enforcing macroscopic constraints, e.g. average energy of gas particles [1], see Fig 1A. The method gained popularity in applied sciences in recent decades primarily as a tool for inference from empirical data, for instance in bird flocking [2], neuronal firing [3], or protein variability [4].

However, realistic biological questions often do not adhere to the assumption of stationarity. Adaptation of populations to spatial and temporal ecological gradients is an example of a complex non-equilibrium processes in which ecological and evolutionary processes interact [5–9]. How can a simple concept of ME be applied to such systems? The most straightforward approach is to use ME on dynamic trajectories, forcing constraints on the dynamical features (Fig 1B). This approach, called maximum caliber (MC), introduced by [10] is suitable for inference of models and their parameters from dynamic data. Comprehensive reviews of MC [11–13] provide multiple examples where the method has been successfully applied to non-stationary biological processes.

While such an inverse approach is useful for understanding temporal data, in many cases data are not available but the dynamics, although often extremely complicated, are known to be accurately described by the Fokker-Planck equation (FPE). The aim of our work is to demonstrate usefulness of a theoretical dimensionality-reduction technique, which approximates possibly many-dimensional stochastic dynamics to a low-dimensional deterministic dynamics of a few key observable quantities (Fig 1C). The approximation combines a static ME ansatz in the FPE equation with a quasi-stationary assumption. We refer to this method as dynamic ME (DME). DME has been used before to solve problems in quantitative genetics [14–16] and independently in cosmology [17–20]. While the problems in quantitative genetics are formulated using a linear FPE and ME uses relative entropy, the applications in physics are formulated using nonlinear FPE and the ME is based on Tsallis entropy. This work studies linear dynamics, characterized by a linear FPE.

The most surprising feature of DME is its accuracy. The method, although derived from the assumption of quasi-stationarity, remains extremely accurate even far-from-equilibrium. Although numerical evidence of the method’s accuracy has been demonstrated in [14–16, 21], explicit analysis is a challenging mathematical problem. This is analogous to problems in reaction kinetics, where reduction of dynamics based on the quasi-stationary assumption is often used but its validity has been shown rigorously only for a few basic systems using singular perturbation [22–24]. In comparison, the accuracy of DME still remains a mystery.

We will address this puzzle by focusing on two processes: the Ornstein-Uhlenbeck (OU) process, and a logistic model of population growth in a continent-island model. The OU process is studied as an example of a dynamics, for which DME gives an exact solution even in a fully general (non-stationary) form, where the two key parameters are functions of time. We derive the exact solution as well as the DME approximation and show that the dynamics are the same. The stochastic island model, as one of the simplest, yet nonlinear, models in population dynamics is studied as a first step to understand more complex population models where ecological processes interact with evolutionary processes. Despite its simple form the DME is no longer exact, and even fails when migration to the island is less than a threshold 1/2. On the other hand, using numerical simulations we show that for migration rate above this threshold the approximation is extremely accurate even far from equilibrium.

## Dynamic maximum entropy

Here we present a dynamic maximum entropy (DME) method to approximate stochastic dynamics by a FPE [14–21]. The method is based on a combination of ME in statistical physics [1], which solves the stationary problem exactly, with a quasi-stationary assumption, as typically used in chemical kinetics [22] to reduce the number of equations. The method applies to stochastic dynamics with an explicit stationary distribution, even though its application is not limited to such problems (as shown in [21] a solution ansatz, which is not based on the stationary form can sometimes lead to more accurate approximation).

DME was introduced in population genetics to understand how quantitative traits change in time in the presence of various evolutionary mechanisms without resolving details about the dynamics of the underlying gene frequencies. However, the origins reach back to [25] studying genetic algorithms for finding the low-energy state in the Ising spin glass by an analogy with statistical mechanics. The problem reduced to an infinite-dimensional dynamics of cumulants, which were solved numerically for the truncated system. Later, the authors of [26] derived the cumulant dynamics for the population genetics problem with population under selection, mutation and drift. To close the infinite-dimensional dynamical system the authors used perturbation theory in the weak selection regime and separation of time scales in the weak mutation regime. The problem of obtaining closed macroscopic dynamics was systematically resolved in [14] by introducing the DME approximation for the dynamics of quantitative traits under directional selection, mutation and drift. The low-dimensional macroscopic dynamics were obtained by considering suitable macroscopic variables, consistent with the ME solution of the stationary problem, and by using the local equilibrium approximation. The method was further applied to a polygenic trait under stabilizing selection, mutation and drift in [27] and its limitations in the small-mutation regime were resolved in [16] by distinguishing the bulk and the boundary layers of the microscopic distributions of allele frequencies. Although accuracy of the method is still not rigorously understood, the authors of [21] showed an exponential convergence to equilibrium in the FPE by finding a positive spectral gap and investigated an alternative formulation of the DME, which is not exact in the stationary case, but leads to a lower error in the dynamical situation and avoids the problems with small mutation rate.

Independent use of the method in statistical physics focused on exact and approximate solutions of a nonlinear FPE arising in cosmology and in other areas of physics. We traced the first relevant connection to [28], where a relationship was established between the linear Fokker-Planck equation and maximum entropy problem, which uses the generalized Tsallis entropy by finding a family of linear FPEs, whose stationary solutions solve the ME problem with the Tsallis entropy. The use of the generalized entropy led to further studies of problems, mathematically described by nonlinear Fokker-Planck equations. Namely, the authors of [20] found particular time-dependent solutions of the nonlinear FPE with linear drift (generalization of the Ornstein-Uhlenbeck process) and power-law noise, which are solutions of the ME problem with the Tsallis entropy. Their ideas were further developed in [29], introducing an approximate ME approach for the study of the nonlinear FPE, based on the Tsallis entropy, called nonextensive maximum entropy approach. This approximation is analogous to the DME approximation, although the class of problems, as well as the definition of the entropy differs.

Although in most of the previous work the derivation of the DME method was presented phenomenologically, a rigorous derivation of the approximation can be found in [21]. Here we provide a comprehensive summary of the method.

Assume stochastic dynamics with a potential in the Langevin form

$$\begin{array}{c} dx_{k}\left(t\right)=\frac{g^{2}\left(x_{k}\right)}{2}\frac{\partial }{\partial x_{k}}[\sum_{i=1}^{d}\alpha_{i}\left(t\right)A_{i}\left(x\right)]dt+g\left(x_{k}\right)d\xi_{k}\left(t\right)\;. \end{array}$$ (1)

for **x** = (*x*_1_, …, *x*_*N*_), *x*_*k*_ ∈ Ω_*X*_, *t* > 0, and **x**(0) = **x**_0_. The potential in the first term (in the brackets) is a linear combination of time-dependent forces *α*_*i*_(*t*), acting on functions *A*_*i*_(**x**), which may introduce coupling between equations. Function *g*(*x*_*k*_) represents amplitude of stochastic fluctuations and *ξ*_*k*_(*t*) are independent Wiener processes. Previous studies (e.g. [14–16, 27]) focused on examples in population genetics where *x*_*k*_ corresponds to the frequency of a certain gene, affecting some quantitative trait. This frequency depends on evolutionary processes, e.g. selection, mutation, and inherent stochastic fluctuations, described by the forces *α*_*i*_(*t*). The distribution *u*(*t*, **x**) follows dynamics described by the FPE

$$\begin{array}{c} \frac{\partial u(t,x)}{\partial t}=-\sum_{k=1}^{N}\sum_{i=1}^{d}\alpha_{i}\left(t\right)\frac{\partial }{\partial x_{k}}[\frac{g^{2}\left(x_{k}\right)}{2}\;\frac{\partial A_{i}\left(x\right)}{\partial x_{k}}\;u\left(t,x\right)]+\frac{1}{2}\sum_{k=1}^{N}\frac{\partial^{2}}{\partial x_{k}^{2}}[g^{2}\left(x_{k}\right)u\left(t,x\right)]\;, \end{array}$$ (2)

which can be also expressed in the flux form $\partial_{t}u\left(t,x\right)=-\sum_{k=1}^{N}\partial_{x_{k}}J_{k}\left[t,x\right]$. This FPE is complemented with no-flux boundary conditions *J*_*k*_[*t*, **x**] = 0 at *x*_*k*_ ∈ ∂Ω_*X*_ and the initial condition *u*(0, **x**) = *u*_0_(**x**).

### Stationary solution and ME

If the vector of forces $\alpha \left(t\right)=\left(\alpha_{1}\left(t\right),\ldots ,\alpha_{d}\left(t\right)\right)\in R^{d}$ is time-independent then at large times the dynamics approach a stationary distribution, parametrized by the vector of limiting forces

$$\begin{array}{c} {\bar{u}}_{\alpha }\left(x\right)=\frac{1}{Z_{\alpha }}(\prod_{k=1}^{N}\frac{1}{g^{2}\left(x_{k}\right)})\text{exp}[\sum_{i=1}^{d}\alpha_{i}A_{i}\left(x\right)] \end{array}$$ (3)

with the normalization coefficient (i.e., the partition function)

$$\begin{array}{c} Z_{\alpha }=\int_{\Omega_{X}^{N}}(\prod_{k=1}^{N}\frac{1}{g^{2}\left(x_{k}\right)})\text{exp}[\sum_{i=1}^{d}\alpha_{i}A_{i}\left(x\right)]dx\;, \end{array}$$ (4)

where **A** = (*A*_1_, …, *A*_*d*_) is a vector function of the state variables **x** which the forces *α*_*i*_ act on. Using the terminology of statistical physics we refer to functions *A*_*i*_ as observables, as their expectations in problems in physics provide a macroscopic description of the system in terms of its natural observable quantities (i.e., average energy of a gas particle, as formulated by [1]). We extend our scope from looking at a problem with constant forces ***α*** to time-dependent forces ***α***(*t*) to account for realistic scenarios where the dynamics, initially settled to a stationary solution, are pushed out-of equilibrium by changes in the forces ***α***. The dynamics of the expectation 〈*A*_*j*_〉 follows from the FPE. Using notation $B_{ji}=\langle \sum_{k=1}^{N}\frac{g(x_{k})}{2}\frac{\partial A_{j}}{\partial x_{k}}\frac{\partial A_{i}}{\partial x_{k}}\rangle$, $V_{j}=\langle \sum_{k=1}^{N}g^{2}\left(x_{k}\right)\frac{\partial^{2}A_{j}}{\partial x_{k}^{2}}\rangle$ we obtain

$$\begin{array}{ccc} \frac{\partial }{\partial t}\left\langle A_{j}\right\rangle & = & \sum_{i=1}^{d}B_{ji}\alpha_{i}+\frac{1}{2}V_{j}\;. \end{array}$$ (5)

This forms a system of ordinary differential equations for 〈**A**〉, which is generally not closed due to nonlinearity of the functions *A*_*j*_(**x**). Next we define a logarithmic relative entropy

$$\begin{array}{c} H\left[u|{\bar{u}}_{\alpha }\right]≔-\int_{\Omega_{X}^{N}}u\text{ln}\;\frac{u}{{\bar{u}}_{\alpha }}dx\;, \end{array}$$ (6)

where *u*(*t*, **x**) is a solution of the FPE at time *t* ≥ 0 and *x*_*k*_ ∈ Ω_*X*_. For any *t* ≥ 0 relative entropy in Eq 6 has a maximum at ***α*** = α*, which can be obtained by solving a set of first-order conditions

$$\begin{array}{c} 0=\frac{d}{d\alpha_{i}}H\left[u|{\bar{u}}_{\alpha }\right]=\int_{\Omega_{X}^{N}}\frac{u}{{\bar{u}}_{\alpha }}\frac{d}{d\alpha_{i}}{\bar{u}}_{\alpha }dx={\left\langle A_{i}\right\rangle }_{u}-{\left\langle A_{i}\right\rangle }_{{\bar{u}}_{\alpha }}\;. \end{array}$$ (7)

The above intriguing relationship states that if for a given time *t* there exists a maximum of the relative entropy in Eq 6 with respect to all *α*_*i*_ reached for some choice of parameters α*, then the expectation of *A*_*i*_ through the distribution *u*(*t*, **x**) equals the expectation through the stationary distribution ${\bar{u}}_{\alpha^{*}}\left(x\right)$ at this time. This simple fact, shown previously in [21] and in a slightly different form in [14–16] suggests that instead of the full representation of the problem using FPE one could trace only the *d*-dimensional dynamics of α*, that parametrize the approximate solution by the form in Eq 3. Furthermore, the two representations agree in terms of the expectations 〈*A*_*i*_〉 at a given time. Concavity of the relative entropy is implied by the following relationship

$$\begin{array}{ccc} \frac{d^{2}}{d\alpha_{i}d\alpha_{j}}H\left[u|{\bar{u}}_{\alpha }\right] & = & -\int_{\Omega_{X}^{N}}[\frac{d}{d\alpha_{j}}\text{ln}{\bar{u}}_{\alpha }][\frac{d}{d\alpha_{i}}\text{ln}{\bar{u}}_{\alpha }]udx+\int_{\Omega_{X}^{N}}[\frac{1}{{\bar{u}}_{\alpha }}\frac{d^{2}}{d\alpha_{i}d\alpha_{j}}{\bar{u}}_{\alpha }]udx\\ & = & -\text{Cov}{\left(A_{i},A_{j}\right)}_{{\bar{u}}_{\alpha }} \end{array}$$ (8)

analogous to similar expressions in [14–16, 21] stating that the Hessian of the relative entropy is positive semidefinite. Note that the solvability of the Eq 7 (for α*) follows from Eq 8 when the covariance matrix is positive definite [21] (it is always positive semidefinite).

Note that the DME can be also defined using the relative entropy, which compares the distribution *u* with the reference distribution *ϕ*, where *ϕ* is a solution of the problem in the absence of forces ***α***. This approach was used in the population genetic applications where *ϕ* represented the neutral distribution in the absence of selection and mutation [14, 16, 27]. ME is then formulated as a constrained optimization where each force ***α***_*i*_ (evolutionary/ecological) enters the problem in the form of the Lagrange multiplier, corresponding to a constraint on a specific complementary macroscopic quantity **A**_*i*_. The alternative formulation of the ME leads to the same mathematical outcome as the one presented in this section. Moreover, it allows us to keep some of the forces constant throughout the evolutionary timescales considered by including them in the reference distribution, while others, which are dynamically adapting, added through the constraints.

### Dynamical approximation

We have established a relationship between the solution of the full stochastic dynamics in Eq 2 and a stationary form *u*_α*_ parametrized by suitable effective forces α* following ME. However, as we demonstrated in Fig 1A, ME is applicable only to static problems. When the system is out-of-equilibrium, we need to establish a dynamic relationship between the values α*(*t*_1_) and α*(*t*_2_) for *t*_1_ ≠ *t*_2_ by using the information captured by the FPE.

To derive the DME approximation of Eq 2 we use an ansatz $u\left(t,x\right)={\bar{u}}_{\alpha (t)}\left(x\right)+R\left(t,x\right)$ for some continuous ***α***(*t*) where *R*(*t*, **x**) is the time-dependent residual. The dynamics of the expectations in Eq 5 become

$$\begin{array}{c} \frac{\partial }{\partial t}{\left\langle A\right\rangle }_{\alpha }=B_{\alpha }\alpha \left(t\right)+\frac{1}{2}V_{\alpha }+[B_{R}\alpha \left(t\right)+\frac{1}{2}V_{R}-\frac{\partial }{\partial t}{\left\langle A\right\rangle }_{R}]\;. \end{array}$$ (9)

where 〈⋅〉_*u*_ represents expectation through distribution *u* and ${\langle f\left(x\right)\rangle }_{\alpha }≔{\langle f\left(x\right)\rangle }_{{\bar{u}}_{\alpha (t)}}$. Now we make two key assumptions. First, we assume that the residual terms in the bracket of Eq 9 are small and we neglect them. In addition, we also impose a quasi-stationarity approximation, assuming that α* are chosen to satisfy the equilibrium relationship

$$\begin{array}{c} B_{\alpha^{*}}\alpha^{*}\left(t\right)+\frac{1}{2}V_{\alpha^{*}}=0 \end{array}$$ (10)

for all *t* > 0. Both steps are easy to justify if the forces ***α***(*t*) are slowly changing (in the adiabatic regime) and the solution of the FPE is thus close to an equilibrium form in Eq 3. However, its validity when out-of-equilibrium is not clear. We use Eq 10 to replace **V**_***α***_ by **V**_α*_ and **B**_***α***_ by **B**_α*_ in Eq 9 to approximate

$$\begin{array}{c} \frac{\partial }{\partial t}{\left\langle A\right\rangle }_{\alpha^{*}}\approx B_{\alpha^{*}}\left(\alpha \left(t\right)-\alpha^{*}\right)\;. \end{array}$$ (11)

Finally, to obtain a closed dynamical system for α* we use the chain rule, noting that $\frac{\partial {\langle A\rangle }_{\alpha^{*}}}{\partial t}=\frac{\partial {\langle A\rangle }_{\alpha^{*}}}{\partial \alpha^{*}}\frac{\partial \alpha^{*}}{\partial t}$. Combination of Eqs 7 and 8 imply that differentiation of an expectation 〈*A*_*i*_〉_α*_ with respect to *α*_*j*_ gives us a covariance **C**_α*_ ≔ Cov(*A*_*i*_, *A*_*j*_)_α*_. Therefore

$$\begin{array}{c} \frac{\partial \alpha^{*}}{\partial t}=C_{\alpha^{*}}^{-1}B_{\alpha^{*}}\left(\alpha \left(t\right)-\alpha^{*}\right)\;,\;\alpha \left(0\right)=\alpha_{0} \end{array}$$ (12)

together with a parametric form in Eq 3 represent DME approximation of dynamics in Eq 2. Solution of Eq 12 can be plugged into the stationary parametric form in Eq 3, which allows us to not only study the accuracy of the key moments used in DME, but also to compute any statistical feature of the approximate solution and compare it with the exact solution.

Note that Eq 12 can be solved for any prescribed continuous function ***α***(*t*) and in the most extreme case, the forces ***α***(*t*) can contain step changes, which clearly violate the quasi-stationarity assumption. However, all previously studied applications of DME approach showed that the approximation captures extremely well the expectations of the key functions even when the forces change rapidly. This is one of the most remarkable and unexpected features of the DME approach which will be studied here.

Realistic situations (e.g. selection acting on quantitative traits that depend on many alleles) typically involve high-dimensional stochastic dynamics with nonlinearities and coupling terms [14–16]. However, for simplicity we consider examples leading to a simpler one-dimensional form with a remark that more complex models can be analyzed using this approach as long as the stationary distribution of the FPE is explicit. However, even when **x** is a scalar **A** and ***α*** are vectors in all problems studied here: these vectors summarise the infinite-dimensional distribution of **x**.

## Ornstein-Uhlenbeck process

### The model

The importance of examples where the DME becomes exact may lead us to the understanding of why the approximation works even for more general cases. In the area of nonlinear FPE such an example was provided by [20] who analyzed a stochastic process with a linear advection and power-law noise, i,e, a generalization of the Ornstein-Uhlenbeck process. They showed that the nonextensive maximum entropy method is exact for the studied process.

Here we outline a simple example of stochastic dynamics where DME reproduces the exact dynamics, namely the standard OU process with linear relaxation to an equilibrium in the presence of constant Gaussian noise (examples include a particle under friction, animal motion, financial time series, etc.). The presentation in this section is partly pedagogical, although we are not aware of other works where the OU process is studied within the context of DME. The state variable *x* in the OU process dynamically adapts to the value *μ* at a speed given by *β*, further affected by a white noise of a magnitude *σ*

$$\begin{array}{c} dx=\beta (\mu -x)dt+\sigma d\xi (t)\;. \end{array}$$ (13)

We consider *β*(*t*) and *μ*(*t*) to be time-dependent, with long-time limits *β*_∞_ and *μ*_∞_ and unless otherwise stated we consider *σ*(*t*) = *σ*_0_ to be constant. Note that *σ*(*t*) can be replaced by a constant using transformation of time in Eq 13 (when *σ*(*t*) is smooth and invertible) without changing the character of the problem. The stationary distribution of the Eq 13 can be obtained from the FPE, which describes time-evolution of the probability distribution of *x*, denoted by *u*(*t*, *x*)

$$\begin{array}{c} \frac{\partial }{\partial t}u=-\frac{\partial }{\partial x}[\beta (t)(\mu (t)-x)u]+\frac{\sigma_{0}^{2}}{2}\frac{\partial^{2}}{\partial x^{2}}u\;, \end{array}$$ (14)

by letting *t* → ∞. Defining

$$\begin{array}{c} {\bar{u}}_{\alpha }\left(x\right)=\frac{1}{Z}\text{exp}[\frac{1}{\sigma^{2}}\alpha \cdot A]\;, \end{array}$$ (15)

this yields

$$\begin{array}{c} {\bar{u}}_{\alpha_{\infty }}\left(x\right)=\frac{1}{Z}\text{exp}[\frac{2\mu_{\infty }\beta_{\infty }}{\sigma_{0}^{2}}x-\frac{\beta_{\infty }}{\sigma_{0}^{2}}x^{2}]=\frac{1}{Z}\text{exp}[\frac{1}{\sigma_{0}^{2}}\alpha_{\infty }\cdot A]\;, \end{array}$$ (16)

where $Z=\text{exp}\left(\mu_{\infty }^{2}/\sigma_{0}^{2}\right)\sqrt{2\pi \sigma_{0}^{2}/\beta_{\infty }}$ is the normalization factor, ***α***_∞_ = (2*μ*_∞_ *β*_∞_, −*β*_∞_) and **A** = (*x*, *x*^2^). If the initial condition in Eq 13 is the stationary distribution corresponding to the parameters (*β*_0_, *μ*_0_, *σ*_0_), i.e., as a Gaussian $x_{0}∼N\left(\mu_{0},\sigma_{0}^{2}/2\beta_{0}\right)$, then the solution of Eq 14 is a Gaussian N(m(t),v(t)) for every *t* ≥ 0. This well-known result can be found for instance in [30, 31]. The general form of this solution is *u*(*x*, *t*) = *f*(*t*)exp[−*g*(*t*)(*x* − *h*(*t*))^2^] where the functions *f*, *g*, *h* satisfy the following dynamical system

$$\begin{array}{cc} \frac{df}{dt} & =f\left(\beta \left(t\right)-\sigma_{0}^{2}g\right)\;,\;f\left(0\right)=\frac{\beta_{0}}{\sqrt{2\pi \sigma_{0}^{2}}}\;, \end{array}$$ (17)

$$\begin{array}{cc} \frac{dg}{dt} & =2g\left(\beta \left(t\right)-\sigma_{0}^{2}g\right)\;,\;g\left(0\right)=\beta_{0}/\sigma_{0}^{2}\;, \end{array}$$ (18)

$$\begin{array}{cc} \frac{dh}{dt} & =\beta \left(t\right)\left(\mu \left(t\right)-h\right)\;,\;h\left(0\right)=\mu_{0}\;. \end{array}$$ (19)

Note that the last equation is just the deterministic version of the OU process. In the special case when the coefficients *β*(*t*) = *β*_∞_ and *μ*(*t*) = *μ*_∞_ for all *t* > 0 while *β*_∞_ ≠ *β*_0_, *μ*_∞_ ≠ *μ*_0_ and *σ*_0_ are constants the mean *m*(*t*) and the variance *v*(*t*) solve the system of ODEs

$$\begin{array}{cc} \dot{m} & =\beta_{\infty }\left(\mu_{\infty }-m\right)\;,\;\dot{v}=\sigma_{0}^{2}-2\beta_{\infty }v\;, \end{array}$$ (20)

with initial conditions *m*(0) = *μ*_0_ and $v\left(0\right)=\frac{\sigma_{0}^{2}}{2\beta_{0}}$. This system has an explicit solution

$$\begin{array}{cc} m(t) & =\mu_{\infty }+\left(\mu_{0}-\mu_{\infty }\right)e^{-\beta_{\infty }t}\;, \end{array}$$ (21)

$$\begin{array}{cc} v(t) & =\frac{\sigma_{0}^{2}}{2\beta_{0}}e^{-2\beta_{\infty }t}+\frac{\sigma_{0}^{2}}{2\beta_{\infty }}\left(1-e^{-2\beta_{\infty }t}\right)\;, \end{array}$$ (22)

which satisfies *m*(*t*)→*μ*_∞_ and $v\left(t\right)\to \sigma_{0}^{2}/2\beta_{\infty }$ as *t* → ∞.

The stationary solution ${\bar{u}}_{\alpha_{\infty }}\left(x\right)$ in Eq 16 solves a variational ME problem with the relative entropy defined in Eq 6 using forces ***α***_∞_ and observables **A**. Even though the OU process has three natural parameters (*β*, *μ*, *σ*) ME implies that the stationary solution is a 2-parameter family of functions of form in Eq 16, due to the fact that time-dependence in *σ*(*t*) can be removed by transforming time in the dynamics. This allows us to keep the volatility of the process fixed in the DME approach.

We briefly outline the key steps in the derivation of DME. The forces and observables are ***α***(*t*) = (2*μ*(*t*)*β*(*t*), −*β*(*t*)) and **A** = (*x*, *x*^2^). In the following calculations we assume temporal dependence of ***α*** but we suppress its notation. The expectations 〈*A*_*i*_〉 follow a closed system of ODEs

$$\begin{array}{c} \frac{d}{dt}\left\langle A\right\rangle =(\begin{array}{cc} 1/2 & \left\langle x\right\rangle \\ \left\langle x\right\rangle & 2\langle x^{2}\rangle \end{array})\alpha +(\begin{array}{c} 0\\ \sigma_{0}^{2} \end{array})=B\alpha +V\;. \end{array}$$ (23)

To apply the DME approximation we assume that at every time there are effective forces α* = (2μ* β*, −β*) such that **B**_α*_ α* + V* = 0 (with the moments in matrix **B**_α*_ evaluated at the stationary distribution ${\bar{u}}_{\alpha^{*}}$). Using the stationarity condition V* = −**B**_α*_ α* to substitute **V** by V* and **B** by **B**_α*_ in Eq 23 we obtain

$$\begin{array}{c} \frac{d}{dt}\left\langle A\right\rangle =B_{\alpha^{*}}\left(\alpha -\alpha^{*}\right)\;. \end{array}$$ (24)

In essence, DME approximates the general solution of Eq 14 with the Gaussian initial condition by a stationary form ${\bar{u}}_{\alpha^{*}\left(t\right)}$ with time-dependent parameters α*(*t*). The matrix **B**_α*_ can be expressed in terms of the effective forces α*, although we will write most of the expressions in terms of μ* and β* (the transformation from α* to (μ*, β*) is regular). The matrix **B**_α*_ can be expressed as

$$\begin{array}{c} B_{\alpha^{*}}=(\begin{array}{cc} 1/2 & \mu^{*}\\ \mu^{*} & 2{\left(\mu^{*}\right)}^{2}+\sigma_{0}^{2}/\beta^{*} \end{array})\;. \end{array}$$ (25)

Finally, we change variables in Eq 24 using the scaled covariance matrix ${\left\{C_{\alpha^{*}}\right\}}_{ij}=\text{Cov}\left(A_{i},A_{j}\right)/\sigma_{0}^{2}$ to obtain dynamics of α*

$$\begin{array}{ccc} C_{\alpha^{*}} & = & \frac{d{\left\langle A\right\rangle }_{\alpha^{*}}}{d\alpha^{*}}=\frac{1}{2\beta^{*}}(\begin{array}{cc} 1 & 2\mu^{*}\\ 2\mu^{*} & \sigma_{0}^{2}/\beta^{*}+4{\left(\mu^{*}\right)}^{2} \end{array})\;, \end{array}$$ (26)

$$\begin{array}{ccc} C_{\alpha^{*}}^{-1} & = & \frac{2\beta^{*}}{\sigma_{0}^{2}}(\begin{array}{cc} 4\beta^{*}{\left(\mu^{*}\right)}^{2}+\sigma_{0}^{2} & -2\mu^{*}\beta^{*}\\ -2\mu^{*}\beta^{*} & \beta^{*} \end{array})\;. \end{array}$$ (27)

Plugging this into Eq 24 leads to

$$\begin{array}{cc} \frac{d\alpha^{*}}{dt} & =C_{\alpha^{*}}^{-1}B_{\alpha^{*}}\left(\alpha -\alpha^{*}\right)=2\beta^{*}(\begin{array}{cc} \frac{1}{2} & -\mu^{*}\\ 0 & 1 \end{array})(\begin{array}{c} 2\beta \mu -2\beta^{*}\mu^{*}\\ \beta^{*}-\beta \end{array})\;. \end{array}$$ (28)

After some algebraic manipulation we obtain a 2-dimensional dynamical system for the effective forces α*

$$\begin{array}{cc} \frac{d}{dt}\left(2\mu^{*}\beta^{*}\right) & =2\beta^{*}\left(\mu \beta -\mu^{*}\beta^{*}\right)+2\mu^{*}\beta^{*}\left(\beta -\beta^{*}\right)\;, \end{array}$$ (29)

$$\begin{array}{cc} \frac{d}{dt}\left(-\beta^{*}\right) & =2\beta^{*}\left(\beta^{*}-\beta \right)\;. \end{array}$$ (30)

This coupled dynamical system can be transformed into decoupled dynamics of μ* and β* of the form

$$\begin{array}{ccc} \frac{d\mu^{*}}{dt} & = & \beta \left(t\right)(\mu \left(t\right)-\mu^{*})\;, \end{array}$$ (31)

$$\begin{array}{ccc} \frac{d\beta^{*}}{dt} & = & 2\beta^{*}\left(\beta \left(t\right)-\beta^{*}\right)\;. \end{array}$$ (32)

First, note that the dynamical system Eqs 31 and 32 is independent of the noise magnitude *σ*_0_, which is considered constant. This is expected and follows from the same scaling of the variance of the OU process (exact or DME approximation) and $\sigma_{0}^{2}$. For the exact OU process, this scaling can be confirmed for instance by the dynamics of the variance in the form $\frac{dv}{dt}=-2\beta \left(t\right)v+\sigma_{0}^{2}$ derived from Eq 14. On the other hand, the DME solution has an explicit Gaussian form ${\bar{u}}_{\alpha^{*}\left(t\right)}$, introduced in Eq 15, which leads to the same scaling between its variance and $\sigma_{0}^{2}$ provided β*(*t*) is independent of *σ*_0_.

The dynamical system in Eqs 31 and 32 is explicitly solvable since it is decoupled and while the first equation is linear, the second one is logistic. The DME solution is consistent with Eqs 18 and 19 with the relationship between the dynamic variables *h*(*t*) = μ*(*t*) and $\sigma_{0}^{2}g\left(t\right)=\beta^{*}\left(t\right)$. The first two moments of the distribution are given by *m*(*t*) = μ*(*t*) and $v\left(t\right)=\sigma_{0}^{2}/\left(2\beta^{*}\left(t\right)\right)$, which are functions of effective forces α*. This is due to the linearity of the OU process, which yields a closed dynamics of the first two moments and thus preserves a Gaussian form of the solution at each time, provided we started with a Gaussian initial condition. Note that the OU process is not the only stochastic process where DME provides an exact solution. As [18] showed, a nonlinear extension of the OU process can be solved exactly using a ME ansatz.

### Numerical example

**Fig 2:**
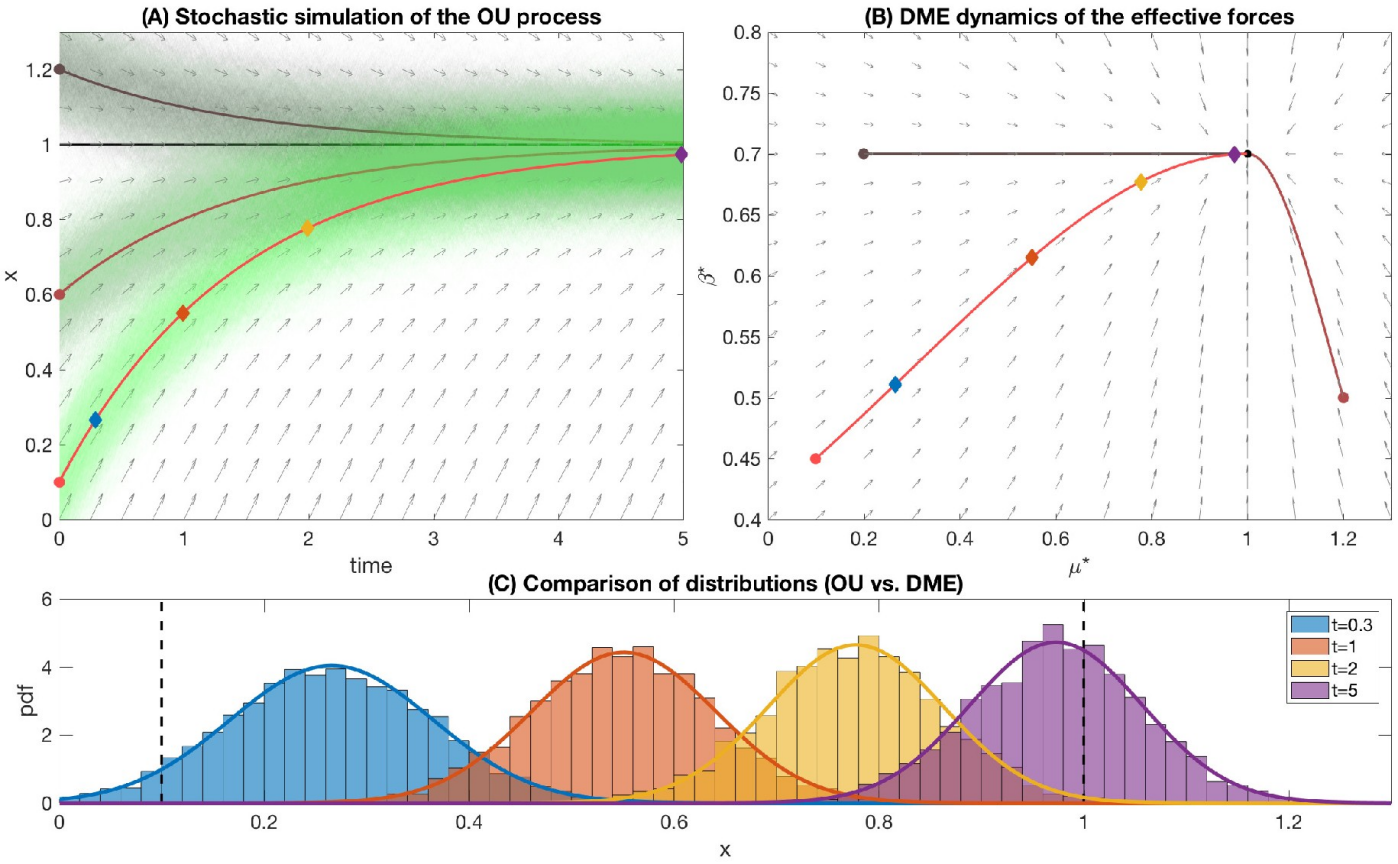
Numerical example of the OU process. (A) Numerical simulations of the OU process with parameters *β*_∞_ = 0.7, *μ*_∞_ = 1, *σ*_0_ = 0.1. We used three random initial conditions from a distribution $N(x_{0},\sigma_{0})$ with *μ*_0_ = *x*_0_ = 1.2, 0.6, 0.1 and *β*_0_ = 0.7, 0.5, 0.45. (B) Effective forces (μ*, β*) following dynamics in Eqs 31 and 32 corresponding to the same set of initial conditions as in panel A. (C) Histograms of *x*(*t*) at times *t* = 0.3, 1, 2, 5 (initial condition *β*_0_ = 0.45, *μ*_0_ = 0.1, *σ*_0_ = 0.1 as in panel A) from the simulated data and approximated distributions in Eq 16 for the effective forces. The time points correspond to the diamonds of matching color in the panels A-B. Code in S1 Code.

Fig 2A shows a numerical simulation of the OU process for three choices of initial Gaussian distribution (centered at *x*_0_ = 0.1, 0.6, 1.2) for 3000 trajectories for each case (all parameters summarized in the figure legend). Initially, the system is in a stationary state corresponding to parameters *μ*_0_, *β*_0_, *σ*_0_ (Gaussian form with parameters $x_{0},\;\sigma_{0}^{2}/2\beta_{0}$). However, at *t* = 0 the parameters of the OU process rapidly change, pushing the system out-of-equilibrium. As a response, the distribution of sample trajectories follows a Gaussian form at each time, eventually converging to $N(\mu_{\infty },\sigma_{0}^{2}/2\beta_{\infty })$. In Fig 2B we plot the 2-dimensional DME dynamics of effective forces β*, μ*, which is exact. Each trajectory (the vector field of the dynamical system is plotted as well) represents the complete solution of the FPE for a given parameter choice (at each time it is a Gaussian). The microscopic distribution at four different times in panel C shows an agreement between stochastic simulations (histograms) and microscopic distributions obtained from the DME approach (solid curves). In general, the goal of DME is to approximate the dynamics on the macroscale, thus, we do not expect DME to capture also the microscopic distribution. However, for the OU process DME is exact and thus the method recovers both the macroscale and the microscale properties of the process without loss of precision.

## Dynamics of a single island with immigration

Here we use the DME method to approximate stochastic population dynamics. We will study a simple, yet nonlinear model—stochastic logistic population growth in a single island with immigration from other habitats.

### The model

In the case of unlimited resources and in the absence of predation, populations would grow indefinitely. However, in natural populations this is not the case: various factors impose bounds on this exponential growth. The logistic growth model describes population size regulation in the absence of demographic stochasticity (i.e., when the population size is infinite). At low population sizes, when resources are abundant and competition is low, the population grows with its intrinsic growth rate, *r*. However, the total growth rate of the population decreases linearly with increasing population size. In particular, the growth rate is zero when the population is at carrying capacity, *K*, when each individual replaces itself in each generation. The carrying capacity represents the maximal sustainable population size. We follow the population in a single island, with a migration from other habitats at rate *m*. In the presence of demographic stochasticity the described population dynamics can be formulated using a stochastic differential equation

$$\begin{array}{c} dn=[n(r-\lambda n)+m]dt+\sqrt{\gamma n}d\xi \;, \end{array}$$ (33)

where *n*(*t*) represents the population size at time *t*, λ = *r*/*K* is the density regulation and *γ* describes the variance in population size. For the sake of simplicity we fix *γ* = 1 corresponding to a Poisson(1) number of offspring for each individual (with total variance *n*). Extinction in this stochastic dynamics for *m* = 0 is unavoidable from a mathematical point of view (as the process is a critical branching process) but can be prevented by migration when *m* > 0.

In general, complex eco-evolutionary interaction requires including changes in population size due to demographic processes, changes occurring in gene frequencies due to selection, as well as the various feedback mechanisms connecting them. Feedback loops between population sizes and gene frequencies can result from migration and hard selection, i.e. when the size of the population depends on its genetic composition. Such questions were studied in [32], but only in a stationary case. Relaxing the assumption of stationarity makes the problem more realistic, and inherently more difficult. The stochastic logistic dynamics with immigration in Eq 33, despite its simplicity, serves as the first step to understanding the biologically more realistic scenario of stochastic models in population dynamics [33]. We are interested in how changes in the environment reflect the dynamics of biological quantities, particularly when the system is out of equilibrium. Since the model is nonlinear, the dynamics of moments, i.e., average population size, etc., are not closed. Nevertheless, the DME method can be applied to reduce the stochastic dynamics to a low-dimensional deterministic dynamics of the key observables.

Based on Eq 33, we find the corresponding FPE describing the time evolution of the probability distribution *u*(*t*, *n*) (which is of the same form as Eq 2):

$$\begin{array}{c} \frac{\partial u}{\partial t}=-\frac{\partial }{\partial n}[(n(r-\lambda n)+m)u]+\frac{1}{2}\frac{\partial^{2}}{\partial n^{2}}\left[nu\right]. \end{array}$$ (34)

The stationary solution of Eq 34 can be found in the form of a potential function. Note that it indeed has the same form as the distribution that we observed earlier in Eq 3 to maximize entropy:

$$\begin{array}{c} u\left(n\right)=\frac{1}{Z}\frac{1}{n}\text{exp}\{2(rn-\frac{\lambda n^{2}}{2}+m\;\text{log}\left(n\right))\}=\frac{1}{Z}v\left(n\right)e^{2\alpha \cdot A}, \end{array}$$ (35)

where $v\left(n\right)=\frac{1}{n}$ is the baseline distribution (the stationary solution without any forces acting on the system), $A=(n,-\frac{n^{2}}{2},\text{log}\left(n\right))$ is a set of observables, and ***α*** = (*r*, λ, *m*) is a set of the ecological forces driving the system. The potential function ***α*** ⋅ **A** consists of the effects of growth, density regulation, and migration. We assume that migration is strong (*m* > 1/2) so even though the function *v*(*n*) is not integrable on Ω_*X*_ = (0, ∞), the function *u*(*n*) is both integrable and bounded for small population sizes *n*. The expectations of the observables have biologically meaningful interpretations, and can, in principle, be measured. In our case, 〈*n*〉 corresponds to the expected population size, 〈*n*^2^〉 to the second moment of population size, and the third term, 〈log(*n*)〉 is the logarithm of the geometric mean of the population size. The normalizing constant Z, which is the function of the effective forces *α*, plays an important role, as a generating function for quantities of interest [14]

$$\begin{array}{c} \frac{\partial \text{log}(Z)}{\partial (2\alpha_{j})}=\left\langle A_{j}\left(n\right)\right\rangle ,\;\frac{\partial^{2}\text{log}\left(Z\right)}{\partial {\left(2\alpha_{i}\right)}^{2}}=\text{Cov}\left(A_{i}\left(n\right),A_{j}\left(n\right)\right)=C_{i,j}. \end{array}$$ (36)

Given a set of forces ***α***, the system evolves to a stationary distribution in Eq 35 that maximizes entropy with constraints on the observables, where 2***α*** serve as the Lagrange multipliers. We are interested in how the dynamics change when the set of forces changes in time, and in the most extreme case when the set of initial forces ***α***_0_ change rapidly to a new set of values ***α***_1_. The observables will evolve towards the new stationary state, which creates a path between ***α***_0_ and ***α***_1_ in the space of effective forces.

Under the diffusion approximation we can derive ordinary differential equations (similarly to Eq 5 for the changes in the mean of the observables $A=(n,-\frac{n^{2}}{2},\text{log}\left(n\right))$

$$\begin{array}{cc} \frac{d}{dt}\left\langle n\right\rangle & =r\left\langle n\right\rangle -\lambda \left\langle n^{2}\right\rangle +m, \end{array}$$ (37)

$$\begin{array}{cc} \frac{d}{dt}[-\frac{\left\langle n^{2}\right\rangle }{2}] & =-(m+\frac{1}{2})\left\langle n\right\rangle -r\left\langle n^{2}\right\rangle +\lambda \left\langle n^{3}\right\rangle , \end{array}$$ (38)

$$\begin{array}{cc} \frac{d}{dt}\left\langle \text{log}\left(n\right)\right\rangle & =r-\lambda \left\langle n\right\rangle +(m-\frac{1}{2})\langle \frac{1}{n}\rangle , \end{array}$$ (39)

where the choice of **A** follows from the stationary form in Eq 35. Eqs 37–39 can be written using the matrix notation:

$$\begin{array}{cc} \frac{d}{dt}\left\langle A\right\rangle & =(\begin{array}{ccc} \left\langle n\right\rangle & -\langle n^{2}\rangle & 1\\ -\langle n^{2}\rangle & \left\langle n^{3}\right\rangle & -\langle n\rangle \\ 1 & -\langle n\rangle & \left\langle \frac{1}{n}\right\rangle \end{array})\alpha +(\begin{array}{c} 0\\ -\frac{1}{2}\left\langle n\right\rangle \\ -\frac{1}{2}\langle \frac{1}{n}\rangle \end{array})=B\alpha +V. \end{array}$$ (40)

This dynamical system is not closed, yet, we may apply the DME method to derive a 3-dimensional approximation for the dynamics of effective forces α* of the form in Eq 12 with a particular form of matrices **B**_α*_ and **C**_α*_. To do this, we approximate the elements of **B** and **V** using the stationary approximation **B**_α*_ α* + V* = 0. Substituting **V** = −**B**_α*_ α* into Eq 40 and using that **B** ≈ **B**_α*_, we obtain $\frac{\partial \langle A_{i}\left(n\right)\rangle }{\partial t}\approx \sum_{j}B_{i,j}^{*}\left(\alpha_{j}-\alpha_{j}^{*}\right)$. Change of variables yields

$$\begin{array}{c} \frac{d\alpha^{*}}{dt}={\left[\frac{\partial {\left\langle A\right\rangle }_{\alpha^{*}}}{\partial \alpha^{*}}\right]}^{-1}\frac{d{\left\langle A\right\rangle }_{\alpha^{*}}}{dt}=C_{\alpha^{*}}^{-1}B_{\alpha^{*}}\left(\alpha -\alpha^{*}\right). \end{array}$$ (41)

The expectations of various functions of variable *n* appearing in matrices **B**_α*_ and **C**_α*_ can be expressed analytically under the condition that the migration rate is not too low ($m>\frac{1}{2}$). Let us call the *k*^th^ moment of the stationary distribution *G*(*k*), this can be expressed analytically in terms of hypergeometric functions (suppressing * notation)

$$\begin{array}{cc} G(k) & =\int_{0}^{\infty }n^{k}v\left(n\right)e^{2\alpha A}=\int_{0}^{\infty }n^{k+2m-1}\text{exp}\left\{-\lambda n^{2}+2rn\right\}=\frac{1}{2}\lambda^{\frac{1}{2}\left(-1-k-2m\right)}\times \end{array}$$ (42)

$$\begin{array}{cc} & \times (\sqrt{\lambda }\Gamma (\frac{k}{2}+m){\;}_{1}F_{1}(\frac{k}{2}+m,\frac{1}{2},\frac{r^{2}}{\lambda })+ \end{array}$$ (43)

$$\begin{array}{cc} & +2r\Gamma (\frac{k+1}{2}+m){\;}_{1}F_{1}(\frac{k+1}{2}+m;\frac{3}{2};\frac{r^{2}}{\lambda })), \end{array}$$ (44)

if Re(*k* + 2*m*/*γ*)>0. Using the function *G*, all the moments of interest can be expressed as

$$\begin{array}{c} G\left(0\right)=Z,\;\frac{G(1)}{G(0)}=\left\langle n\right\rangle \;,\;\frac{G(2)}{G(0)}=\left\langle n^{2}\right\rangle \;,\;\frac{G(3)}{G(0)}=\left\langle n^{3}\right\rangle \;,\;\frac{G(-1)}{G(0)}=\langle \frac{1}{n}\rangle \;. \end{array}$$ (45)

Thus

$$\begin{array}{cc} B_{\alpha^{*}} & =\frac{1}{G(0)}(\begin{array}{ccc} G(1) & -G(2) & G(0)\\ -G(2) & G(3) & -G(1)\\ G(0) & -G(1) & G(-1) \end{array})\;. \end{array}$$ (46)

Furthermore, we can express 〈log(*n*)〉 analytically by taking the *j*^*th*^ derivative of *G*(*k*) with respect to *m*:

$$\begin{array}{c} H\left(k,j\right)=\left\langle n^{k}\;\text{log}{\left(n\right)}^{j}\right\rangle =\frac{1}{2^{j}}\frac{\partial^{\left(j\right)}G_{k}}{\partial m^{j}}. \end{array}$$ (47)

The covariance matrix of the observables evaluated at the quasi-stationary distribution parametrized by the effective forces can then be written as

$$\begin{array}{c} C_{\alpha^{*}}=(\begin{array}{ccc} \frac{G(2)}{G(0)}-\frac{G{\left(1\right)}^{2}}{G{\left(0\right)}^{2}} & \frac{1}{2}(\frac{G(1)G(2)}{G{\left(0\right)}^{2}}-\frac{G(3)}{G(0)}) & \frac{H(1,1)}{H(0,0)}-\frac{G(1)H(0,1)}{G(0)H(0,0)}\\ \frac{1}{2}(\frac{G(1)G(2)}{G{\left(0\right)}^{2}}-\frac{G(3)}{G(0)}) & \frac{1}{4}(\frac{G(4)}{G(0)}-\frac{G{\left(2\right)}^{2}}{G{\left(0\right)}^{2}}) & \frac{1}{2}(\frac{G(2)H(0,1)}{G(0)H(0,0)}-\frac{H(2,1)}{H(0,0)})\\ \frac{H(1,1)}{H(0,0)}-\frac{G(1)H(0,1)}{G(0)H(0,0)} & \frac{1}{2}(\frac{G(2)H(0,1)}{G(0)H(0,0)}-\frac{H(2,1)}{H(0,0)}) & \frac{H(0,2)}{H(0,0)}-\frac{H{\left(0,1\right)}^{2}}{H{\left(0,0\right)}^{2}} \end{array})\;. \end{array}$$ (48)

The 3-dimensional nonlinear dynamical system in Eq 41 along with Eqs 46 and 48 defines the DME approximation of the island model. Note that the method is fully general and may be applied for arbitrary functions ***α***(*t*), capturing non-stationary ecological situations.

### Failure of the DME method for small migration rate

An apparent failure of the DME for the island model arises in the parameter regime *m* < 1/2. The stationary form, which is used as a parametric ansatz in the DME, behaves for low population sizes as *n*^2*m*−1^ leading to a singularity at *n* = 0 for *m* < 1/2. Even though the population size distribution is integrable, the average 〈*n*^−1^〉, appearing in the matrix B* is unbounded. A similar problem arises when a quantitative trait is studied under selection, mutation and inherent stochasticity when mutation is weak. In [16] we showed how to resolve this problem by splitting the domain of the independent variable to a bulk and the boundary, where the singularity occures. A similar approach can be applied here as well.

### Numerical example

**Fig 3:**
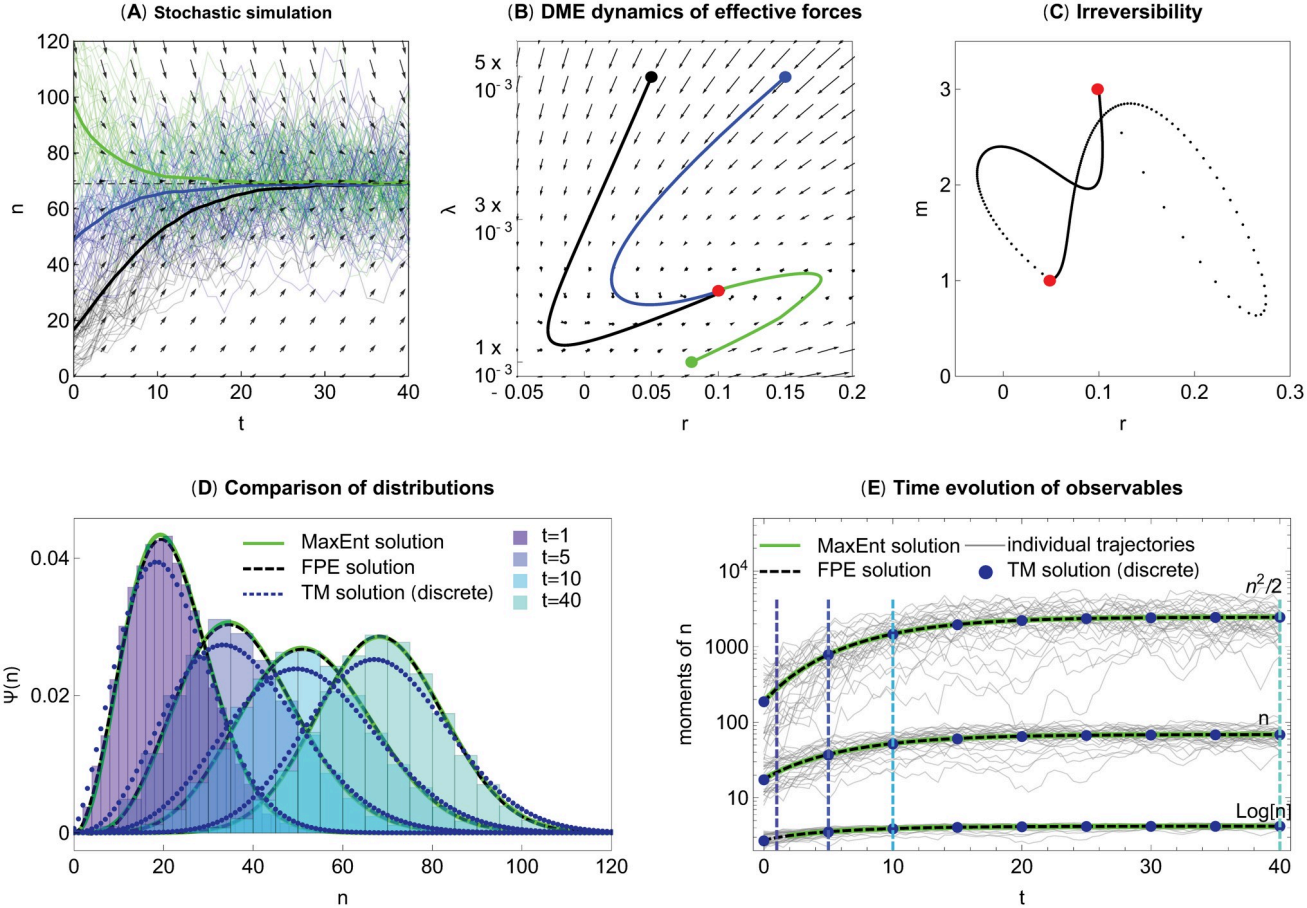
Numerical example of the island model. (A) Numerical simulations of stochastic population dynamics on a single island with immigration. Parameters are *α*_1_ = {*r*, λ, *m*} = {0.1, 0.002, 3}. We used initial conditions, *α*_0,1_ = {0.05, 0.005, 1} (black), *α*_0,2_ = {0.15, 0.005, 5} (blue), and *α*_0,3_ = {0.08, 0.001, 2} (green). (B) Corresponding dynamics of the effective forces projected to the (*r*, λ) space. (C) Irreversibility of the process: 2D projections of the trajectories between *α*_1_ = {0.1, 0.002, 3} and *α*_0,1_ = {0.05, 0.005, 1} and reversed are not the same. (D) Histograms of population sizes at *t* = 1, 5, 10, 40 with initial condition *α*_0,1_ = {0.05, 0.005, 1} (black curves in panels A-B). The numerical solution of the corresponding FPE, the discrete transition matrix prediction, and the DME all show a close match. (E) The three observables *n*, log(*n*), and *n*^2^/2. Code in S1 Code.

To understand the relationship between the dynamics of the original system in Eq 33 and the dynamics of the reduced system we simulated individual population size trajectories using the Euler-Maruyama method and then compared them to the predictions of the DME method. In Fig 3A, we see the simulated trajectories for three sets of initial conditions. The initial conditions were not fixed, but instead were randomly drawn from a stationary initial distribution, parametrized by the growth rate (*r*_0_), the strength of density regulation (λ_0_), and migration (*m*_0_). Starting in equilibrium, we changed the environmental forces abruptly to new values ***α*** = (*r*, λ, *m*) at time *t* = 0. This forced the system out of equilibrium and shifted the trajectories toward the new equilibrium, independent of the initial condition.

Instead of using the original stochastic differential equation, in the DME we follow the dynamics of the effective forces α* = (r*, λ*, m*) as they move to the new equilibrium shown in Fig 3B. Note, that the parameter space is 3 dimensional, but only a 2 dimensional projection is presented here. A single point in this space describes the full distribution of population sizes.

Fig 3C shows that the dynamics of the effective forces in the DME approximation of the stochastic logistic model with migration are irreversible, i.e., the trajectory in the space of effective forces when the system changes from ***α***_0_ to ***α***_1_ is different from changing ***α***_1_ to ***α***_0_. In both cases the system was initialized with the stationary distribution and the forces were changed at time *t* = 0.

How close is the distribution approximated by DME to the real distribution? Despite the simple form of our equations, it is not possible to solve it explicitly analytically. Thus, we compared the numerically computed distributions for the original (i.e., exact) problem with the numerically computed distributions obtained by the DME approximation (in Fig 3D). We used three approximations to solve the original problem: (1) using the full stochastic simulation (Fig 3A, distribution at each time approaches the exact distribution when the time step in the stochastic simulation is small and the number of trajectories is large), (2) numerically solving the FPE for the process by the native solver of *Mathematica*, and (3) using the transition matrix method, where we follow a Markov-chain, a continuous time birth-death process on the discrete space of non-negative integers. On the other hand, we used an Euler scheme to solve the DME system.

Although all methods are approximate, the original problem can be solved with any precision using methods (1–2) and thus we may focus here on the accuracy of the DME method itself. We find that they are in a good agreement with each other, the only exception being the transition matrix method, which is defined on a discrete space (and to retain biological meaning also has a slightly different variance from Eq 33). We compared the distributions at different time points in panel D. We observe that although the transition in the observable quantities is rather slow and monotonic, the changes in the effective forces can be abrupt and non-monotonic. Note, that the DME method does not guarantee that the microscopic population size distributions are identical, in fact, they can differ substantially. Nevertheless, the DME method aims to capture the agreements between the macroscopic variables. Fig 3E shows all three key observables and shows that the DME method is in an excellent agreement with the model.

In the previous example we demonstrated that the method works well in the most extreme case, when the forces in the dynamics change abruptly. This is surprising, as the DME approximation is based on a quasi-stationary assumption, which would intuitively work only when the forces are changing slowly. This may suggest that the method will perform even better under slow environmental changes, which in reality may be more likely to happen than an abrupt change. Temporal differences in the environment can be abrupt, causing populations to become maladapted and possibly drive them to extinction. Less rapid environmental changes can be observed on various timescales, for example the warming of the oceans [34] or the yearly cycle of seasons [35], resulting in different migration rates throughout the year due to varying resource abundance.

**Fig 4:**
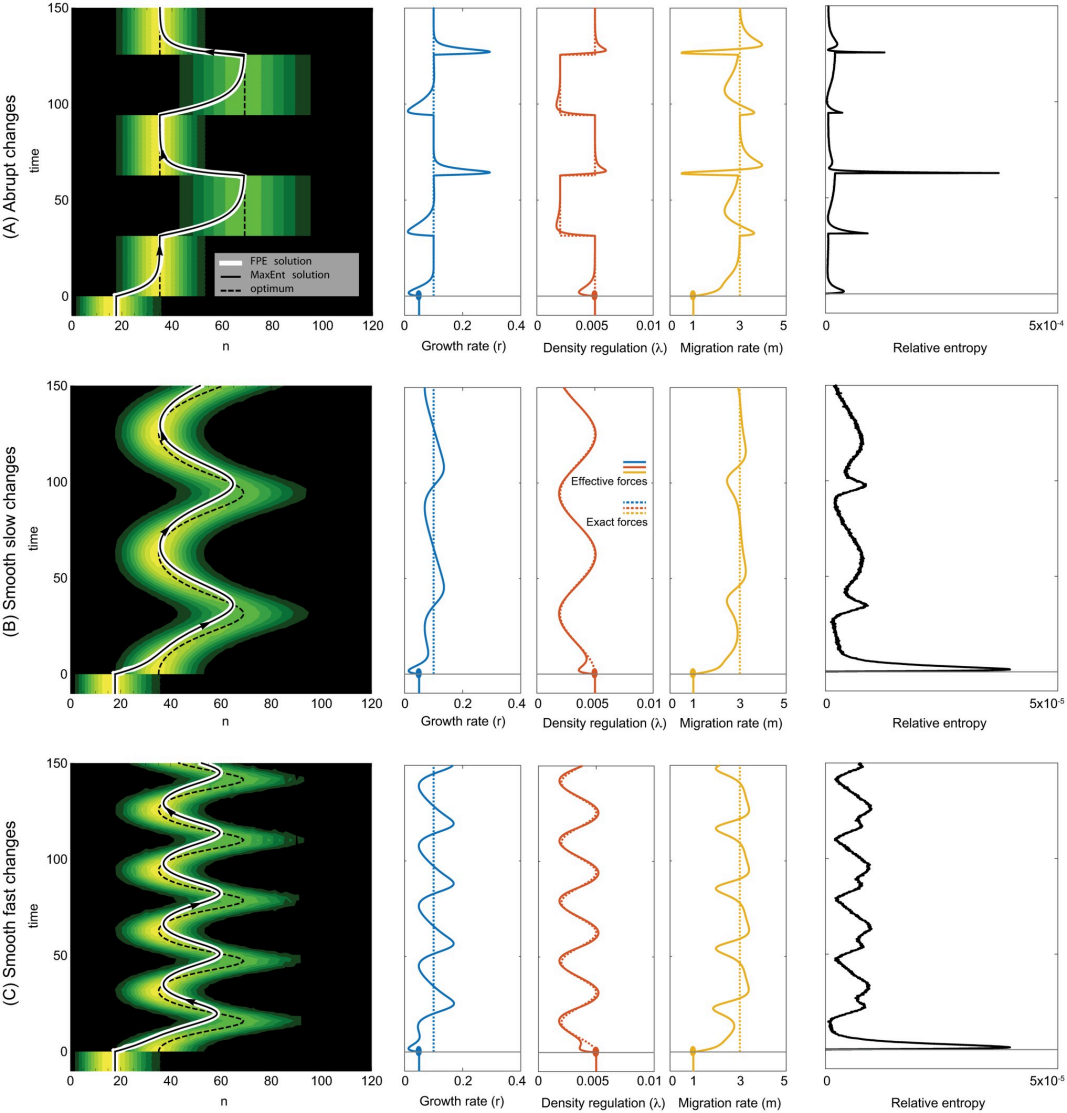
Periodic changes in carrying capacity between 20 and 50. The system starts from equilibrium with parameters {0.05, 0.005, 1} (as in Fig 3), then periodic shifts occur between {0.1, 0.0005, 3} and {0.1, 0.0002, 3} (blue, red, yellow). The equilibrium distribution of population size is shown as it changes in time (background colors). The black dashed line is the mean equilibrium population size, the black solid line shows the solution of the DME, whereas the white is the solution of the FPE. The error is measured by relative entropy, see the Eq 6.

In Fig 4 we compare periodic changes for three scenarios: an abrupt, a slow and a fast but continuous change. We see that the solution of the non-equilibrium dynamics lags behind the equilibrium of the environment, and that the amount of this lag depends on the speed of change of the ecological forces. While the faster environmental oscillations result in a smaller range of average population sizes, the range of effective forces, on the contrary, increases. In the extreme case of very fast environmental changes (e.g. oscillations with large frequency, or environmental temporal noise of a fixed variation) one expects that the average population size will stay almost stationary as the convergence to equilibrium is much slower than the timescale of environmental fluctuations, while the rapid fluctuations will impact the effective forces, similarly to our first scenario in Fig 4A.

We found that the solution of DME is in a good agreement with that of the FPE, with the temporal dynamics in the DME and the moments in the FPE equation being indistinguishable by eye (white or black lines). We quantified the error of the DME approximation by the relative entropy between the exact population density, which solves the FPE equation, and the DME approximation, which is computed from the dynamics of the effective forces. The magnitude of this error is larger in the scenario A where the environmental parameters change abruptly, as compared to their smooth changes in cases B-C (note the diferences in the range between the panels). Surprisingly, even in the first scenario where the environmental parameters change abruptly, and the dynamics have time to adapt to the changed environment, the relative entropy does not decay monotonically during the adaptation period. We observe that after a brief initial exponential drop (showed on a logarithmic scale in S1 Fig), the relative entropy follows a slower non-monotonous pattern. This is likely caused by the dynamics of the effective forces, each of which shows non-monotonous convergence to the new equilibrium at a different time scale. This non-monotonicity of the relative entropy, which we observe in all studied scenarios, makes the problem difficult to study analytically.

## Discussion

We presented an application of the dynamic maximum entropy method, which helps reduce complexity of the stochastic process by linking microscopic quantities to macroscopic observables.

We first studied the Ornstein-Uhlenbeck process with time-dependent parameters. We used the DME method—instead of following the full stochastic dynamics, we followed just the key observables (in this case the first two moments), which change deterministically. The observables and the forces acting on them were identified from the potential form of the stationary solution (in this case Gaussian). The dynamic problem was solved by the DME method, which uses the stationary ansatz with time-dependent parameters to best approximate the dynamics of observables. We derived a two-dimensional dynamical system of the effective forces that characterize the solution of the OU dynamics. We showed that despite the intricacies of the DME approximation, the DME dynamics coincide with an exact solution of the OU process. This is because the dynamical equations for the first two moments are closed and thus the solution is Gaussian at all times. However, even though the OU dynamics are linear, the effective forces solve a nonlinear system of ordinary differential equations.

The focus of our work is on the stochastic island model, represented by nonlinear population dynamics based on a logistic equation supplemented by migration from other islands. The key parameters of the problem, the intrinsic growth rate, the carrying capacity, and the migration rate, are in general functions of time, reflecting temporal environmental changes, which take the problem out-of-equilibrium. Nonlinearity of the process results in the dynamics of moments 〈*n*^*k*^〉 which are not closed for any k. Therefore we used DME to derive a 3-dimensional nonlinear dynamical system for the effective forces using DME. The associated observables are no longer just the first three moments but include 〈*n*〉, 〈−*n*^2^/2〉 and 〈log*n*〉. Unlike for the OU model, the DME approximation of the island model with migration is no longer exact. The system is not fully explicit but contains terms using hypergeometric functions. Nevertheless, it can be solved for dynamic environmental forces using standard numerical solvers.

We found that the effective forces in the DME approximation lag behind the true environmental forces, which is more pronounced when the environmental forces change faster. However, even in cases of rapid changes of the environmental forces the observables in the DME approximation are still extremely accurate at all times and thus the effective forces serve as a proxy for the dynamics. When the environmental forces settle to constant values, the effective forces also converge to these values. The DME serves as a change of optics where we represent the non-equilibrium dynamics using a series of equilibria parametrized by dynamical effective forces. The strength of the DME method in our view is not in speeding up the numerical method for solving the problem but in better understanding the underlying dynamics in an appropriate low-dimensional space.

Although the FP equations give a complete description of how the whole probability distribution evolves through time, it is not feasible to solve them numerically for more than two or three variables, and analytic results become intractible, once one moves away from linear models. The FP equations do not bring much actual understanding, without the help of heuristic approximations such as ME. Although it is possible to use the quasi-stationary approximation without the connection to ME, the method provides us with useful information. By formulating the suitable ME problem for the stationary distribution we learned which macroscopic quantities are important for the low-dimensional projection of the dynamics. More specifically, DME identifies pairs of complementary variables—for example, in the population dynamic example, (*n*, −*n*^2^, *log*(*n*)) correspond to growth rate, strength of density regulation, and migration. While the observables enter the variational problem via the constraints, the forces are the corresponding Lagrange multipliers in ME. This is an intriguing extension to more general nonlinear stochastic processes of an idea familiar from statistical thermodynamics.

In addition, the DME method can be placed into the arsenal of methods for non-equilibrium dynamics, as we have shown in Fig 1. It is built from the stationary ME method but unlike the MC method, which is essentially a ME method applied on temporal trajectories, it uses the FPE to establish relationships between the time points.

One of the most striking properties of the DME method is its accuracy on the macroscopic level. This is surprising because the quasi-stationary assumption suggests validity of the approach only when the applied forces change slowly (i.e., adiabatically). However, even for fast-changing forces the approximation stays very accurate. We have shown numerically that the relative entropy between the exact and approximate solutions does not decay monotonically even in the simplest scenario of an abrupt change in the ecological forces. This along with the unusual form of the DME approximation makes analytical study of the accuracy of the method a difficult mathematical problem which remains open to date, despite insight provided in [21].

The remarkable accuracy of the DME approximation, even when conditions change abruptly, remains mysterious to us. We can draw an analogy with path integration, where the approximation that fluctuations around the most likely path are Gaussian is exactly correct for all cases that can be solved explicitly, and is accurate even with strong nonlinearity [36]. The breakdown of DME when $m<\frac{1}{2}$ is more understandable: then, the concentration of probability near the small population boundary in effect constrains one of the free variables, so that the approximation cannot fit as well, based on the remaining variables. This problem can be resolved by considering boundary layer in the probability distribution, thus effectively increasing the number of free variables, as investigated in detail in [16].

This work outlines the first step towards studying more complex questions where the complexity of the problem is prohibitive for studying the full problem. In particular, our future goal is to explore eco-evolutionary dynamics where the ecological and population genetic timescales interact. Such interaction has been studied in [32] but only in the stationary case. Although the existence of an explicit stationary distribution in principle allows us to explore the dynamics in the non-stationary environment using DME, the structure of the problem poses multiple difficulties that need to be resolved first.

The approach may also be suited to stochastic problems in different disciplines. The method is based on the structure of the problem, in which the stochastic dynamics are described by the FPE and the stationary solution is explicit. This includes a wide range of problems accross disciplines, for example stochastic coagulation-fragmentation dynamics when the rates satisfy a detailed balance condition (existence of an explicit stationary distribution for this problem was shown in [37]).

## Supporting information

S1 Fig The logarithmic error of the DME approximation. The error corresponds to the dynamic scenario in Fig 4A. The dashed gray lines depict the times at which the changes of the ecological forces occur. The exponential decay is highlighted in green.
(TIF)

S1 Code Code in mathematica and Matlab. The enclosed files Matlab_OrnsteinUhlenbeck_matlab.m and Mathematica_IslandWithMigration.nb contain code behind our results. The first file, executable in Matlab, shows implementation of the Ornstein-Uhlenbeck process, its stochastic simulation and the DME method. It returns a figure similar to Fig 2 in our work. The second file, executable in Mathematica, contains en example simulation of the stochastic island model. All parts of the code are supplemented by an explanation and the outcome figures, similar to the figures in the main paper.
(ZIP)
