# Supplementary figures and images for: Dynamic maximum entropy provides accurate approximation of structured population dynamics
Source: PLoS Comput Biol. 2021 Dec 1;17(12):e1009661. doi: 10.1371/journal.pcbi.1009661 (PMC8668141; doi:10.1371/journal.pcbi.1009661)

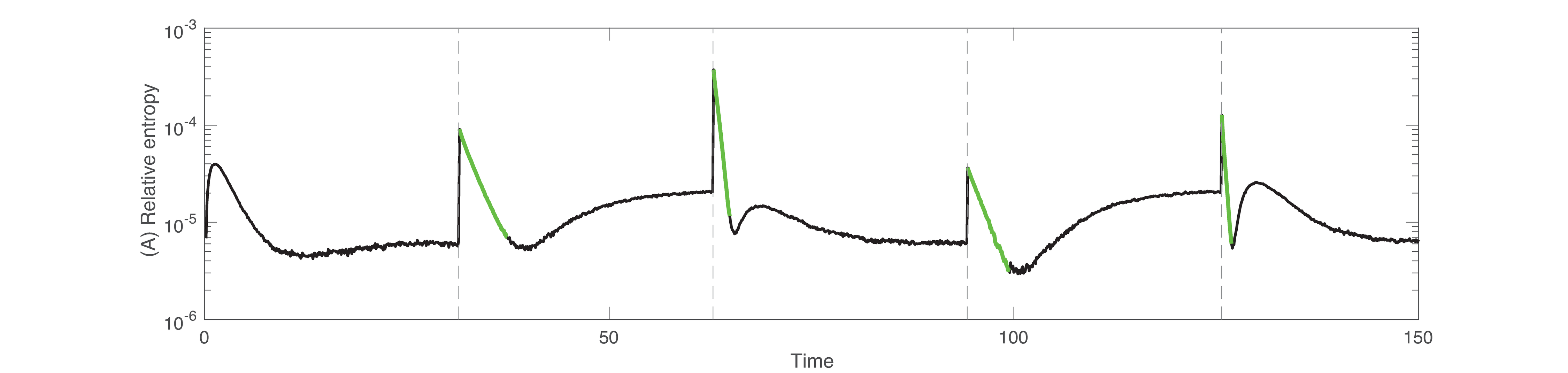

Supplement: S1 Fig — The error corresponds to the dynamic scenario in Fig 4A. The dashed gray lines depict the times at which the changes of the ecological forces occur. The exponential decay is highlighted in green. (TIF) [file pcbi.1009661.s001.tif]
