# Supplementary material for: Dynamic maximum entropy provides accurate approximation of structured population dynamics
Source: PLoS Comput Biol. 2021 Dec 1;17(12):e1009661. doi: 10.1371/journal.pcbi.1009661 (PMC8668141; doi:10.1371/journal.pcbi.1009661)
Supplement: S1 Code — The enclosed files Matlab_OrnsteinUhlenbeck_matlab.m and Mathematica_IslandWithMigration.nb contain code behind our results. The first file, executable in Matlab, shows implementation of the Ornstein-Uhlenbeck process, its stochastic simulation and the DME method. It returns a figure similar to Fig 2 in our work. The second file, executable in Mathematica, contains en example simulation of the stochastic island model. All parts of the code are supplemented by an explanation and the outcome figures, similar to the figures in the main paper. (ZIP) [file pcbi.1009661.s002.zip › SI_code/readme.rtf]

The enclosed files:Matlab_OrnsteinUhlenbeck_matlab.mMathematica_IslandWithMigration.nbcontain code behind our results. The first file, executable in Matlab, shows implementation of the Ornstein-Uhlenbeck process, its stochastic simulation and the DME method. It returns a figure similar to Figure 2 in our work. The second file, executable in Mathematica, contains en example simulation of the stochastic island model. All parts of the code are supplemented by an explanation and the outcome figures, similar to the figures in the main paper.
